# Vigorous physical activity predicts higher heart rate variability among younger adults

**DOI:** 10.1186/s40101-017-0140-z

**Published:** 2017-06-14

**Authors:** Richard May, Victoria McBerty, Adam Zaky, Melino Gianotti

**Affiliations:** Biology Program, Southern Oregon University, 1250 Siskiyou Boulevard, Ashland, OR 97520 USA

**Keywords:** Physical, Activity, Heart, Variability, Autonomic

## Abstract

**Background:**

Baseline heart rate variability (HRV) is linked to prospective cardiovascular health. We tested intensity and duration of weekly physical activity as predictors of heart rate variability in young adults.

**Main body of the abstract:**

Time and frequency domain indices of HRV were calculated based on 5-min resting electrocardiograms collected from 82 undergraduate students. Hours per week of both moderate and vigorous activity were estimated using the International Physical Activity Questionnaire. In regression analyses, hours of vigorous physical activity, but not moderate activity, significantly predicted greater time domain and frequency domain indices of heart rate variability. Adjusted for weekly frequency, greater daily duration of vigorous activity failed to predict HRV indices.

**Conclusions:**

Future studies should test direct measurements of vigorous activity patterns as predictors of autonomic function in young adulthood.

## Background

Heart rate variability (HRV) reflects central regulation of autonomic activity and is linked to current health status and longer-term health outcomes. Baseline measurements of heart rate variability in adulthood, for example, predict subsequent development of hypertension [1]. Baseline HRV, in turn, is impacted by amount and intensity of exercise. In a study of middle-aged civil servants [2], both moderate and vigorous physical activity predicted greater HRV with effects moderated by gender and overweight status.

Among young adults, studies examining effects of exercise interventions on HRV have yielded inconsistent results [3]. A study that tested effects of habitual activity patterns in different age groups reported non-significant effects in young adults [4]. In contrast, a more recent study involving direct measurement of weekly physical activity identified a significant beneficial impact of achieving recommended levels of vigorous physical activity [5]. The present study tests whether self-reported amounts of weekly activity predict HRV among a group of healthy younger adults. The main question addressed is whether total minutes per week of moderate and vigorous physical activity predict higher HRV. Additionally, we test average daily duration of physical activity as a predictor of HRV.

## Main text

### Methods

Participants were recruited from a Biology course at Southern Oregon University during Winter term, 2016. Participants received course credit as incentive for participation and informed consent was obtained for all participants. The study protocol was approved by the Human Subjects Review Board. A total of 115 students agreed to participate and provided electrocardiographic (ECG) recordings. Exclusion criteria included medical conditions associated with altered autonomic function (e.g., arrhythmia and valve defects) and use of psychotropic medication known to impact autonomic function. Participants with previously diagnosed anxiety or depression were also excluded given potentially lasting effects on autonomic function [6].

Electrocardiographic recordings were collected using a BIOPAC MP-36 system (BIOPAC Systems Inc., Galeta, Ca). Three disposable, pre-gelled electrodes were attached with one just inferior to each clavicle and one inferior to the xiphoid process. Participants were instructed to abstain from alcohol or coffee for 8 h prior to recording and to consume only water for 2 h prior to recording. Following 5 min of quiet rest, 5-min recordings were collected with a sample rate of 1000 samples/s. Recordings were made between 0800 and 1030 hours in a temperature-controlled room. Participants were seated and were instructed to breathe normally with eyes closed. Each event series was first detrended using a high-pass digital filter with a 1-Hz (hertz) cutoff. Additional processing involved a first-derivative transform [7] to distinguish R waves against pronounced T waves. Root mean square of successive differences (RMSSD), low-frequency power (LFP; 0.04–0.15 Hz), and high-frequency power (HFP; 0.15–0.40 Hz) were calculated using BIOPAC Student Lab Pro software (BIOPAC Systems, Inc.) and values were natural log transformed to improve normality [8].

Height, weight, and blood pressure were collected following the ECG recording. Participants also completed a series of on-line questionnaires to assess demographic, health, and psychological data. The Perceived Stress scale [9] was administered to estimate stress exposure during the past month and physical activity was assessed using the International Physical Activity Questionnaire (IPAQ) [10]. IPAQ data were used to estimate minutes per week of both vigorous and moderate physical activity.

Physical activity measures were tested as predictors of time and frequency domain indices of heart rate variability. Adjusted models were also tested that included covariates significantly associated with HRV measures. All statistics were calculated using SPSS (version 22).

## Results

Eighty-two participants included in the study had complete data for all measures. The average age of sample was 23.1 years and 62% were female (Table 1). Thirty-five participants were overweight (BMI ≥25) and three reported smoking cigarettes. Sixty-two participants met American Heart Association (AHA) recommendations (>75 min) for weekly vigorous physical activity and 41 participants met AHA recommendations (>150 min) for weekly moderate physical activity [11]. Age was significantly negatively correlated with lnRMSSD (*r =* −0.23; *p =* .04) and with lnHFP (*r =* −0.25; *p =* 0.02) and diastolic blood pressure was negatively correlated with RMSSD (*r =* −0.24; *p =* 0.03). *t* tests revealed lower lnLFP in females (*t =* 2.8; *p =* 0.008). Perceived stress scores were not significantly correlated with HRV indices or with physical activity measures (*p >* 0.38 for all tests).

**Table 1:** Summary data for demographic, physical activity, and physiological measures

Measure	*x̅*	Standard deviation
Age (years)	23.1	5.4
Systolic blood pressure (mmHg)	120.6	10.2
Diastolic blood pressure (mmHg)	73.2	8.8
Body mass index (kg/m^2^)	25.4	0.5
Vigorous activity (minutes per week)	298.5	276.1
Moderate activity (minutes per week)	241.3	298.7
Perceived stress	16.6	5.5
RMSSD (ms)	63.2	48.8
LFP (ms^2^)	269.6	411.0
HFP (ms^2^)	329.2	727.0

In unadjusted regression models, minutes per week engaged in vigorous physical activity significantly predicted greater lnRMSSD, lnLFP, and lnHFP (Table 2). After adjustment for age, gender, and diastolic blood pressure, this relationship remained significant for lnRMSSD and was marginally significant (*p =* 0.08) for lnHFP. In all analyses, moderate physical activity failed to significantly predict HRV measures. Collinearity among predictor variables was assessed using the variance inflation factor (VIF). The VIF was 1.07 for predictors (vigorous activity and moderate activity) in unadjusted regression models. In adjusted models, the VIF values were 1.20 (vigorous activity) and 1.17 (moderate activity). To test whether effects of vigorous physical activity differed between males and females, models were retested that included a gender-by-vigorous activity interaction term. The interaction was non-significant for all models (*p >* 0.60).

**Table 2:** Multiple regression analyses for predictors of heart rate variability

	lnRMSSD^a^ (ms)		lnLFP (ms squared)		lnHFP (ms squared)	
	Model 1	Model 2^b^	Model 1	Model 2^b^	Model 1	Model 2^b^
	*R* ^2^ (0.10)^c^	*R* ^2^ (0.18)	*R* ^2^ (0.07)	*R* ^2^ (0.15)	*R* ^2^ (0.08)	*R* ^2^ (0.14)
Vigorous activity (minutes)	0.34 (*.002*)^d^	0.24 (*0.04*)	0.27 (*0.02*)	.19 (0.10)	0.30 (*0.01*)	0.20 (0.08)
Moderate activity (minutes)	0.04 (0.74)	0.11 (0.31)	−0.22 (0.05)	−.19 (0.10)	0.06 (0.61)	0.13 (0.25)

^a^Root mean square of successive differences

^b^Age, gender, diastolic blood pressure as covariates

^c^Adjusted *R* ^2^

^d^Standardized beta (*p* value)

**Fig. 1:**
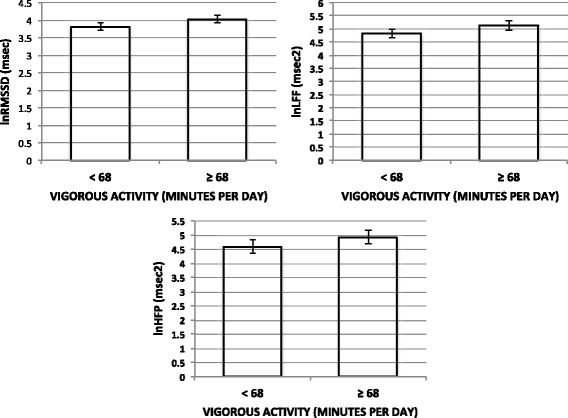
Relationships between daily duration of physical activity and heart rate variability. Bars indicate marginal means (±1 standard error) for **a** lnRMSSD, **b** lnLFP, and **c** lnHFP, adjusted for weekly frequency

An additional goal of the study was to test associations between daily duration of vigorous physical activity and heart rate variability. Bivariate correlations revealed that daily minutes of activity were correlated with lnRMSSD (*r =* 0.35; *p =* 0.001) and lnHFP (*r =* 0.30; *p =* 0.006). When daily duration of vigorous physical activity was coded as higher (<68 min) or lower (≥68 min) based on the sample median, *t* tests indicated significantly greater lnRMSSD (*t =* -2.5; *p =* 0.01) and lnHFP (*t =* -2.1; *p =* 0.04) for subjects in the higher duration group. Since daily duration of vigorous activity was significantly correlated with weekly frequency (*r =* 0.66; *p <* 0.001), analysis of covariance models were used to test differences in HRV indices across daily duration groups, adjusted for days per week of vigorous activity (Fig. 1). In these tests, higher daily minutes of vigorous activity failed to predict lnRMSSD (*F =* 1.4; *p =* 0.23), lnLFP (*F =* 1.1; *p =* 0.29), and lnHFP (*F =* 0.8; *p =* 0.36).

## Discussion

In this study, minutes per week engaged in vigorous physical activity predicted greater time domain and frequency domain measures of HRV. For RMSSD, these effects persisted after adjustment for covariates. Greater daily duration of vigorous physical activity, however, failed to predict HRV when adjusted for weekly frequency. It is unlikely that observed associations were mediated by psychological stress exposure since recent stress was not correlated with HRV measures or with physical activity measures.

Among athletes, age and the intensity or duration of an exercise program are critical influences on HRV [12]. Previous studies have documented positive effects of exercise on RR interval and HF and LF power in aerobically trained younger adults [13] but a non-significant effect of moderate intensity exercise [14]. Physical activity assessed through accelerometry reveals a positive effect on HRV for young adult subjects meeting recommended levels for vigorous activity compared to those meeting recommended levels of moderate activity [5]. In the present study, most subjects were physically active with 76% meeting recommendations for weekly vigorous activity. Within this physically active group of young adults, greater weekly vigorous activity predicted greater HRV indices across the observed range of activity. A question for future research is whether a saturation point is reached [3] beyond which additional exercise no longer increases HRV.

High-frequency and low-frequency components of HRV are thought to reflect distinct neural regulatory mechanisms [15]. While both high-frequency power and time domain indices correlate strongly with pharmacologically measured vagal tone [16], low-frequency power may reflect central modulation of baroreceptor reflexes by both sympathetic and parasympathetic activities [17]. Studies in rodents have suggested mechanisms by which exercise may impact high-frequency and low-frequency components of HRV. These include altered GABA-ergic signaling in the nucleus ambiguous [18] and nucleus of the solitary tract [19]. This study had several limitations that should be noted. Respiration was not controlled for during ECG recording. While respiratory rhythm can impact time and frequency domain indices, especially in certain experimental paradigms [20], recent research suggests that the impact on respiratory sinus arrhythmia is minimal for short-term, resting recordings [21]. In addition, physical activity was assessed by questionnaire and not through direct measurement. Questionnaire-based measures may yield biased estimates of activity, especially for certain populations [22]. Finally, while recent stress exposure was assessed, other psychological factors such as depression that may impact heart rate variability [23] were not.

## Conclusions

Results of the present study are in agreement with previous findings that link vigorous physical activity to higher measures of HRV. Lower levels of physical activity are associated with greater cardiovascular risk with effects mediated in part by autonomic dysfunction [24]. Reduced vagal function in particular is common to multiple risk factors that are predictive of cardiovascular disease [25]. While the present study identified significant effects for self-reported levels of overall activity, future research utilizing more detailed and direct measurements of daily activity could reveal stronger associations with autonomic function in young adulthood.

## Abbreviations

AHA: American Heart Association
ECG: Electrocardiographic
HFP: High-frequency power
HRV: Heart rate variability
Hz: Hertz
IPAQ: International Physical Activity Questionnaire
LFP: Low-frequency power
lnHFP: Natural logarithm of HFP
lnLFP: Natural logarithm of LFP
lnRMSSD: Natural logarithm of RMSSD
ms: Milliseconds
RMSSD: Root mean square of successive differences
